# An exploration of heterogeneity in genetic analysis of complex pedigrees: linkage and association using whole genome sequencing data in the MAP4 region

**DOI:** 10.1186/1753-6561-8-S1-S107

**Published:** 2014-06-17

**Authors:** Shelley B Bull, Zhijian Chen, Kuan-Rui Tan, Julia Poirier

**Affiliations:** 1 Lunenfeld-Tanenbaum Research Institute of Mount Sinai Hospital, 60 Murray Street, Box 18, Toronto, Ontario M5T 3L9, Canada; 2 Dalla Lana School of Public Health, Health Sciences Building, 155 College Street, University of Toronto, Toronto, Ontario M5T 3M7, Canada

## Abstract

We conduct pedigree-based linkage and association analyses of simulated systolic blood pressure data in the nonascertained large Mexican American pedigrees provided by Genetic Analysis Workshop 18, focusing on observed sequence variants in MAP4 on chromosome 3. Because pedigrees are large and sequence data have been completed by imputation, it is feasible to conduct analysis for each pedigree separately as well as for all pedigrees combined. We are interested in quantifying and explaining between-pedigree heterogeneity in linkage and association signals. To this end, we first examine minor allele frequency differences between pedigrees. In some of the pedigrees, rare and low-frequency variants occur at a higher prevalence than in all pedigrees combined. In simulation replicate 1, we conduct variance-components linkage and association analysis of all 894 MAP4 variants to compare analytic approaches in single pedigree and combined analysis. In all 200 replicates, we similarly examine the 15 causal variants in MAP4 known under the generating model. We illustrate how random allele frequency variation among pedigrees leads to heterogeneity in pedigree-specific linkage and association signals.

## Background

Whole genome sequencing holds out the promise of being able to more effectively map the effects of genetic variants on complex traits, and thereby identify the causal variants involved in disease expression [1]. Even when the effect of a causal variant is large, if the allele is rare, the effect size will be overwhelmed in a low-frequency population unless a very large sample can be analysed. As pointed out by Gagnon et al [2] and others, variants that occur rarely in a population are unlikely to segregate in more than a few pedigrees when pedigrees are not ascertained by disease or trait values. However, when a rare allele has entered a family, it can segregate to multiple family members, increasing the allele frequency in that pedigree and improving power to detect rare or low-frequency causal variants.

In analysis of the original Genetic Analysis Workshop pedigree data (reported in detail in Chen et al [3]), we observed substantial between-pedigree heterogeneity and differences between linkage and association tests, which we seek here to explore more thoroughly in the simulated data with the underlying model known. In the original data, pedigree heterogeneity could arise from variation in genetic effect size, in minor allele frequency, or from allelic or locus heterogeneity. In the simulated data, heterogeneity is largely caused by variation in minor allele frequencies between pedigrees, because the causal variants and their effect sizes were fixed under the generating model and applied to all individuals. We anticipated that analysis of the observed sequence data and the simulated systolic blood pressure (SBP) would allow us to describe natural genetic variation among the San Antonio Family Study (SAFS) pedigrees, as well as assess whether selection of pedigrees with linkage can enrich for rare variants and improve detection of variants in association analysis.

## Methods

### SAFS pedigree data

We analyzed the imputed "best guess" sequence genotype data for a total of 959 study participants in the 20 pedigrees, as provided, including 894 MAP4-designated sequence variants encompassing the chromosome 3 region from 47.892183 to 48.130741 megabases (Mb). Under the generating model for the phenotype simulations, the sequence data were the same for each replicate, and the same set of 15 variants was defined as causal, so only the randomly generated phenotypes varied from replicate to replicate. We estimated the minor allele frequency (MAF) at each segregating locus within a pedigree. As described in Chen et al [3], we analyzed the residuals of SBP from censored linear regression, averaged over 3 visits, as a quantitative phenotype accounting for use of antihypertensive medication, age, sex, and smoking.

### Linkage and association analysis

We applied the genetic analysis software SOLAR for variance-components models to assess linkage and association in pedigrees [4]. With single-marker identity-by-descent (IBD) estimates based on kinship and the sequence data for each pedigree, we performed 2-point linkage analysis across the MAP4 region. Association analyses, conducted for each single variant, included measured genotype (MG) analysis, which relates the quantitative trait directly to the genotype in all individuals, and the quantitative transmission disequilibrium test (QTDT), which relates the variation in the quantitative trait to the difference between the genotype observed in the offspring and that expected given the parental genotypes [5]. Association analysis by QTDT is of interest because it is an explicitly pedigree-based association method that detects association in the presence of linkage by testing for transmission disequilibrium. In some sense it is "intermediate" between linkage analysis (purely within-pedigree analysis) and the MG association analysis (purely between individuals). As recommended to reduce type I error [6], we took linkage information into account in the association analysis.

In the QTDT method, the mean phenotype is modelled as a linear combination of fixed effects (ie, genotype score) and random effects (ie, polygenic and linkage components). The genotype scores (*g*) are decomposed into between-family (*b*) and within-family (*w*) components in a fixed-effect model *E*(*phenotype*) = *μ *+ *β*_b_*b *+ *β*_w_*w*. The MG approach estimates regression coefficients with the constraint *β*_b _= *β*_w_. The QTDT approach estimates both *β*_b _and *β*_w _and tests whether the within-family parameter *β*_w _is significantly different from zero. The parameter *β*_w _reflects the within-pedigree correlation between phenotype and the allelic transmission score *w = *(*g-b*) which is the deviation between the observed and expected genotype. It is, therefore, robust to stratification effects [5].

In replicate 1, for each of 894 loci with sequence variants we computed the LOD score, and the asymptotic MG and QTDT p-values for all pedigrees combined and for each pedigree separately, and constructed LOD score and −log10(*p *value) regional profile plots. For processing of all 200 replicates, we considered only the 15 MAP4 causal variants specified in the simulation model. Given the small size of MAP4 relative to a typical linkage region, and the limitations of single-point IBD estimation for linkage analysis, we took the maximum of the 2-point LOD as a regional measure of linkage. We constructed box plots of the LOD score and −log10(*p *value) to examine variation across replicates by pedigree and differences in power among the causal loci.

## Results and discussion

### MAP4 variants in SAFS pedigrees

Of the 894 MAP4 variants, only a fraction was observed within a single pedigree (ranging from 179 in pedigree 47 to 389 in pedigree 5) and as expected, rare variants (MAF <1%) were most prevalent. Overall MAF values for the 15 "causal" loci in MAP4 ranged from 0.5% to 37.8%: 3 common variants (>5% MAF) were observed in all 20 pedigrees, 3 low-frequency variants (1% to 5% MAF) appeared in 7 or more pedigrees, with 9 rare variants (<1% MAF) in 1 to 5 pedigrees. In any one pedigree, only 3 to 8 of 15 variants were observed. When a rare variant was observed, the corresponding pedigree-specific MAF was substantially higher, with nearly all being >1% for causal variants.

### Linkage and association in single pedigrees and all pedigrees combined

**Figure 1:**
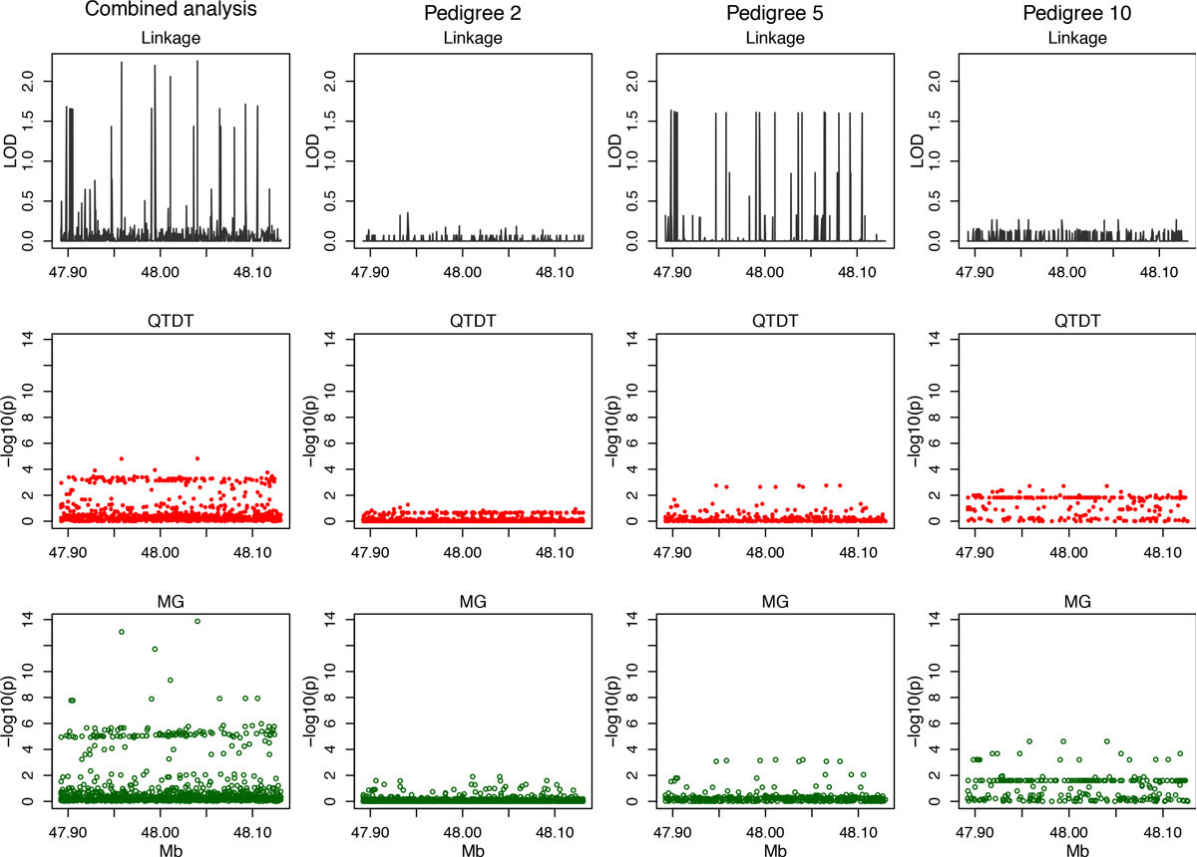
Replicate 1 linkage LOD scores, QTDT and MG association test *p *values across the chromosome 3 MAP4 region (47.89 to 48.13 Mb) for all pedigrees combined and for selected single pedigrees.

In replicate 1, there was marked variation in the combined-pedigree 2-point LOD scores across the MAP4 region (Figure 1). Only pedigree 5 achieved a maxLOD >1.5, and of the remaining pedigrees, all but pedigree 4 had a maxLOD <0.5. In pedigree 5, loci with the high LOD scores also had small MG and QTDT *p *values, whereas pedigree 10, in contrast, had small LOD scores but more significant MG *p *values. The combined pedigree maxLOD of 2.26 and minimum MG asymptotic *p *value of 1.3 × 10^−14 ^occurred at causal locus 10 corresponding to a low-frequency variant (3.2%) with a large effect size and the highest SBP %variance explained within MAP4. At this locus (Table 1), 3 pedigrees with large MAF values had a LOD score >0.25, and the smallest MG and QTDT *p *values (<0.001 and <0.04). At the other extreme, pedigree-specific MG and QTDT analysis failed in pedigrees 4 and 14 which had very low MAF and negligible LOD scores.

**Table 1:** Replicate 1 linkage LOD scores, association *p *values, and regression coefficients for pedigree-specific and combined all pedigree analysis of locus 10 in MAP4

Pedigree	MAF	MAP4 maxLOD	Locus 10 LOD	QTDT *p *value	MG *p *value	QTDT β_w	QTDT β_b	MG β	MG se(β)
2	0.01	0.36	0.06	0.42	**0.012**	−9.2	−29.6	**−21.3**	**8.5**
4	0.02	1.39	0.01	1	1	NA	NA	NA	
5	**0.11**	**1.64**	**1.60**	**0.002**	**6.70E-04**	**−17.4**	**−14.5**	**−14.6**	**3.4**
9	0.12	0.08	0	0.64	0.18	9.0	−13.2	−10.1	7.2
10	**0.07**	**0.27**	**0.26**	**0.002**	**2.40E-05**	**−21.1**	**−19.5**	**−19.5**	**4.2**
11	0.03	0.13	0	1	**0.04**	NA	NA	**−22.5**	**10.6**
14	0.01	0	0	1	1	NA	NA	NA	
16	0.04	0.14	0	1	**0.005**	NA	NA	**−27.6**	**9.3**
20	0.04	0.06	0	1	**0.004**	NA	NA	**−25.8**	**8.8**
21	0.04	0	0	0.98	0.99	0.01	−0.006	NA	10.4
25	0.05	0	0	0.62	0.73	−0.1	0.001	−0.9	7.8
27	**0.06**	**0.35**	**0.32**	**0.04**	**5.40E-04**	**−17.9**	**−36.7**	**−28.2**	**6.0**
									
Combined	0.03		**2.26**	**1.5 × 10^−5^**	**1.3 × 10^−14^**	**−15.9**	**−18.4**	**−17.4**	**2.1**

Location is 48040283 bp with effect size beta = −9.91. SBP %variation explained = 0.028. The variant is segregating in 12/20 pedigrees.

In large pedigrees consisting of multiple nuclear families, the pedigree-specific QTDT analysis yields separate regression coefficients that correspond to between-family and within-family genotype scores calculated in each nuclear family. The between-family score is an expected genotype (typically, an average of the parental genotypes) and the within-family score for an individual is the deviation of their observed genotype from the expected, which is taken as a measure of allelic transmission [5]. Like other transmission-based methods, families in which one or both parents are homozygous can be uninformative for the QTDT test, with a within-family score equal to zero. When a variant occurs infrequently, there will be few heterozygous individuals and a pedigree consisting of multiple such families may be entirely uninformative for estimation of a within-family regression coefficient (as in Table 1: pedigrees 4, 11, 14, 16, 20). In contrast, MG is essentially a linear regression of phenotype on the observed genotype score that includes all individuals in a pedigree, and a combined analysis will also include individuals from pedigrees in which all individuals are homozygous. This is the main reason why QTDT is so much less powerful than MG.

Under the fixed effects part of the QTDT model, the between-family and within-family scores are approximately orthogonal so the between and within regression coefficients are approximately independent [5], but only the latter is robust to population stratification. Because the QTDT within-family regression coefficient in a single pedigree may be based on a small number of informative transmissions, it can be imprecise and the corresponding asymptotic *p *values may be inaccurate, so, as illustrated in Table 1, combined pedigree analysis will be an improvement. In the replicate 1 analysis, the between and within QTDT coefficients agree well for the combined analysis and for the 4 pedigrees with nonnegligible LOD scores, suggesting lack of population stratification bias in the simulated data.

**Figure 2:**
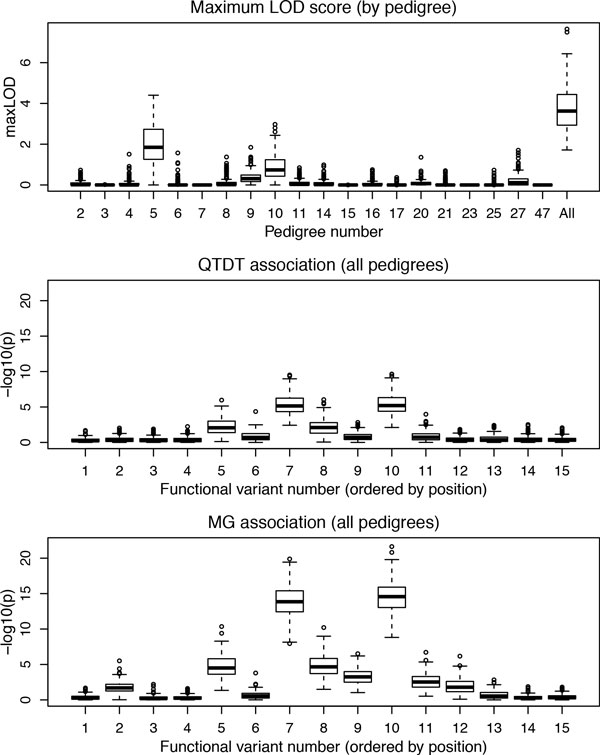
**Box-plot summaries across all 200 replicates of MAP4 maxLOD and association test *p *values for all 15 loci with causal variants in MAP4**.

**Figure 3:**
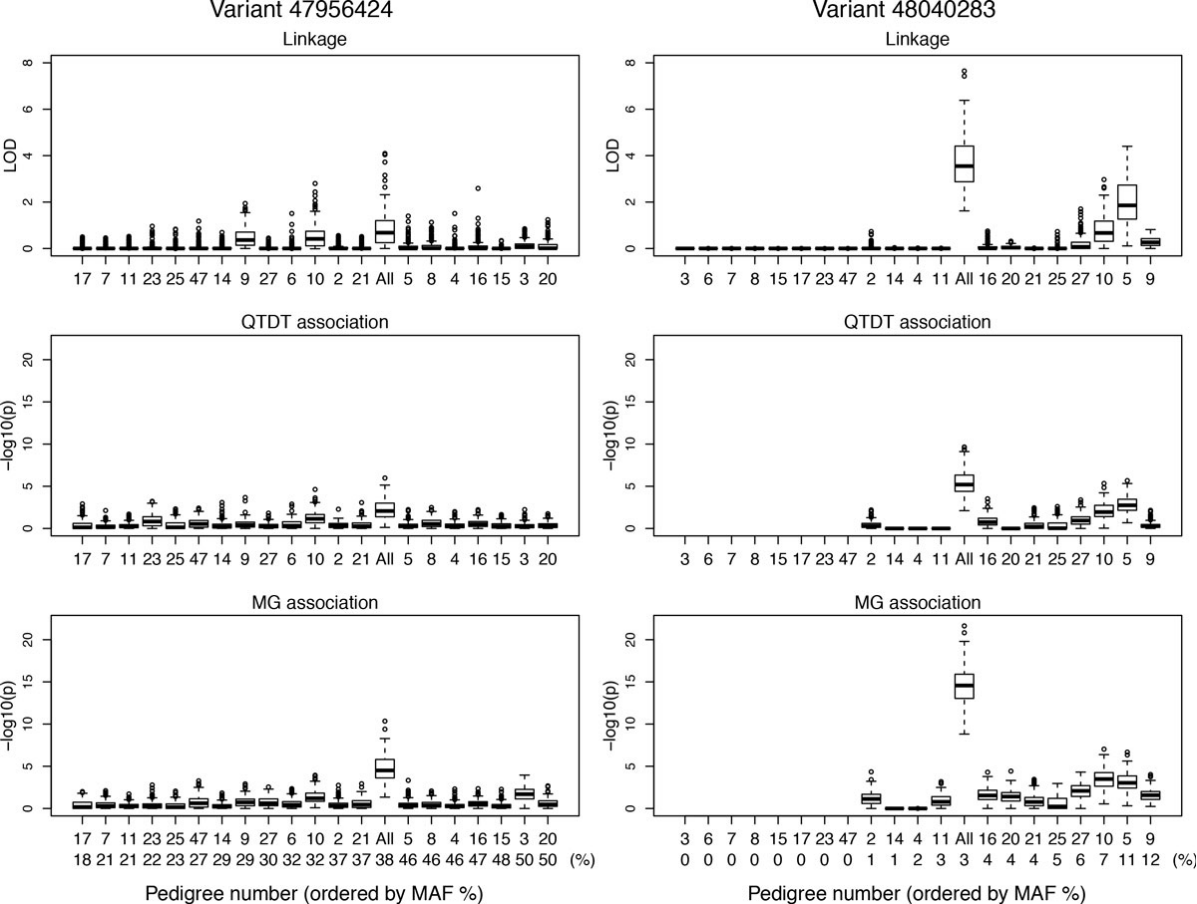
**Box-plot summaries across all 200 replicates of pedigree-specific and combined all pedigree analysis for 2 loci in MAP4**. Linkage LOD scores, QTDT and MG association test *p *values. Pedigrees are ordered on the horizontal axis by pedigree-specific MAF (%). LHS: for variant 5 (47956424 base pairs [bp]), combined pedigree MAF = 37.8%; RHS: for variant 10 (48040283 bp), combined pedigree MAF = 3.2%.

Examination of the distribution of LOD scores and association *p *values across all 200 replicates demonstrates considerable heterogeneity among pedigrees (Figures 2 and 3), driven by differences in the causal variants segregating within a pedigree and the associated variation in MAF. The *p *value distribution profile across loci for QTDT mirrored that for MG, but with substantially reduced significance. Although, as expected, it was difficult to detect association for single rare variants, the common (loci 5, 8, 12) and low-frequency (loci 7, 10, 11) variants were reasonably well-detected by the MG test. Analysis of loci 5 and 8 gave nearly identical results, although only locus 5 had a nonzero effect size under the generating model, reflecting an indirect effect of an underlying haplotype. Loci 7 and 10 were similarly subject to complete linkage disequilibrium within pedigrees; each had independent effects, but these were not distinguishable; the locus 10 regression coefficients (see Table 1) appear to be capturing the combined effects of both. Our analysis did not determine the extent to which the sequence data imputation may have contributed to this linkage disequilibrium. Combined pedigree analysis appeared to be always more powerful than analysis of any single pedigree, for both linkage and association, with the caveat that we did not assess whether the use of asymptotic *p *values was valid.

## Conclusions

Consideration of the combined pedigree maxLOD across the MAP4 region was sufficient to identify a gene-specific region for sequence analysis, even with single-point IBD estimation. For each of the 9 MAP4 rare variants (MAF <1%) included in the phenotype-generating model, segregation of the variant was observed in 5 or fewer of the 20 pedigrees, with mean MAF >1% in the subset. The low-frequency variant responsible for the largest SBP %variation explained (locus 10), which segregated in 12 pedigrees, exhibited the best power for association with good agreement between MG and QTDT signals. Pedigrees with some evidence for linkage at this locus were enriched for the low-frequency variant (or conversely, linkage evidence was contributed according to the frequency of the variant), and it followed that these pedigrees were also more informative in the within-family QTDT assessing transmission disequilibrium, and contributed to smaller standard errors in the MG regression analysis. As we understand it, the Genetic Analysis Workshop 18 (GAW18) pedigrees comprise a sample of large Mexican American families, not ascertained on the phenotype. Therefore differences in MAF between pedigrees arise from random differences in the genotypes of the founder individuals for each pedigree. Because the genetic model used to generate the phenotype data in the simulated data sets is the same in each pedigree, variation in MAF among the pedigrees is a major source of heterogeneity in the pedigree-specific linkage and association tests.

## Competing interests

The authors declare that they have no competing interests.

## Authors' contributions

SBB and ZC designed the overall study and drafted the manuscript. ZC, JP, and KRT conducted statistical analyses. All authors read and approved the final manuscript.
